# The Feasibility and Utility of a Personal Health Record for Persons With Dementia and Their Family Caregivers for Web-Based Care Coordination: Mixed Methods Study

**DOI:** 10.2196/17769

**Published:** 2020-06-26

**Authors:** Colleen M Peterson, Jude P Mikal, Hayley R McCarron, Jessica M Finlay, Lauren L Mitchell, Joseph E Gaugler

**Affiliations:** 1 Division of Health Policy & Management School of Public Health University of Minnesota Minneapolis, MN United States; 2 Division of Epidemiology & Community Health School of Public Health University of Minnesota Minneapolis, MN United States; 3 Institute for Social Research University of Michigan Ann Arbor, MI United States; 4 Center for Care Delivery & Outcomes Research Minneapolis VA Health Care System & University of Minnesota Minneapolis, MN United States

**Keywords:** Alzheimer disease, technology, disease management, personal health record, family caregiving, informal caregiving, caregiver burnout, web-based intervention, assistive technology

## Abstract

**Background:**

Managing the complex and long-term care needs of persons living with Alzheimer disease and related dementias (ADRD) can adversely impact the health of informal caregivers and their care recipients. Web-based personal health records (PHRs) are one way to potentially alleviate a caregiver’s burden by simplifying ADRD health care management

**Objective:**

This study aimed to evaluate Personal Health Record for Persons with Dementia and Their Family Caregivers (PHR-ADRD), a free web-based information exchange tool, using a multiphase mixed methods approach.

**Methods:**

Dementia caregivers (N=34) were surveyed for their well-being and perceptions of PHR-ADRD feasibility and utility at 6 and 12 months using close- and open-ended questions as well as a semistructured interview (n=8). Exploratory analyses compared participants’ characteristics as well as PHR-ADRD use and experiences based on overall favorability status.

**Results:**

Feasibility and utility scores decreased over time, but a subset of participants indicated that the system was helpful. Quantitative comparisons could not explain why some participants indicated favorable, neutral, or unfavorable views of the system overall or had not engaged with PHR-ADRD. Qualitative findings suggested that technology literacy and primary care provider buy-in were barriers. Both qualitative and qualitative findings indicated that time constraints to learn and use the system affected most participants.

**Conclusions:**

Development and dissemination of PHRs for family caregivers of persons with ADRD should aim to make systems user-friendly for persons with limited time and technological literacy. Establishing health care provider buy-in may be essential to the future success of any PHR system.

## Introduction

### Background

In 2018, more than 16 million caregivers provided unpaid care to an estimated 5.8 million Americans living with Alzheimer disease and related dementias (ADRD) [1]. Population projections suggest that this need for assistance will expand as the number of persons aged 65 years and older with ADRD is projected to increase to 13.9 million in the United States by 2060 [2]. Unpaid spouses, children, and significant others assist with a host of complex needs, including basic daily care, symptom management, and care coordination [1]. The stress of providing this extensive care can lead to physical and mental health problems, burnout, and subsequent diminished care quality provided to persons living with dementia [3-7].

A range of interventions have been introduced to alleviate the adverse outcomes of dementia caregiving [1,2]. These interventions include programs to provide training in the management of dementia-related symptoms, bolstering resources through social support coordination, and respite designed to help caregivers maximize time free from care responsibilities [8-10]. More recently, interventions to alleviate caregiver burden and stress have leveraged modern technologies [11-13]. These technologies tend to focus on care recipients, including robotics for help with daily tasks [14,15] and socialization [16,17], remote devices aimed at promoting living at home with in-home monitoring devices [18,19], telemedicine [20,21], and other assistive technologies such as facial recognition software [22].

There is growing recognition of the need for assistive technology for caregivers of persons with dementia as well [13,23]. A number of cost-effective web-based options have emerged to support family ADRD caregivers, such as tailored education and resource portals [24-27]. Internet-based interventions are a low-cost mechanism to present education and provide support. A recent systematic review identified that the most successful internet-based interventions were multicomponent, tailored, and often involved contact with other caregivers as well as guidance from a coach. This resulted in improved decision making and self-efficacy and reduced depression and burden [28].

### Objective

Personal health record (PHR) systems are a form of caregiver-focused technology leveraged to address prolonged and often-fragmented ADRD care needs. PHR systems take advantage of electronic health records (EHRs; or digital records of health information usually maintained at a care provider’s institution) by consolidating information across institutions and offering easier access via web portals accessible by patients or, with permission, family members. Thus, the use of PHRs is a promising avenue for more effective coordination of ADRD health information between informal caregivers and health care professionals (eg, primary care physicians and case managers) and subsequent improved chronic disease management [29-32].

There remains little guidance regarding PHR systems or features that best support the complex and individualized care coordination needs of dementia caregivers. Personal Health Record for Persons with Alzheimer Disease or Related Dementia and Their Family Caregivers (PHR-ADRD), a web-based care coordination tool, aimed to fill this gap.

## Methods

### Overview

This study examines the use of PHR-ADRD—a free web-based care coordination tool. A multiphase parallel convergent mixed methods pilot (QUAN+QUAL → QUAL) tested the feasibility and utility of the PHR-ADRD system to assist family caregivers in managing information and care during the course of ADRD [33]. Phase I used a different web platform and informed recruitment and health information access strategies for phase II [34], which is the focus of this analysis. This evaluation study of phase II aims to help elucidate the gap between development and implementation and successful adoption of care coordination tools such as PHR-ADRD among individuals providing care to persons with dementia.

### Personal Health Record for Persons With Dementia and Their Family Caregivers Development

#### Phase I

The development of PHR-ADRD proceeded through 2 phases. In phase I, participants (N=13) tested the feasibility of Microsoft HealthVault, a similar PHR-ADRD as that used in phase II. HealthVault is a portal with manual entry or linkages with partnering health care providers for merging patient health care records into one profile, accessible anywhere via an internet-enabled device. Free features comparable with the PHR-ADRD platform used in phase II included maintaining basic demographics (eg, sex and blood type); health provider notifications; medicine and potential interaction information; as well as medical procedures performed, test results, and health condition histories [34].

Phase I revealed that a crucial barrier to the use of a PHR is access to health information from providers. Providers denied requests for information because a patient signature was illegible, refused to accept signatures from the person designated as having power of attorney, and frequently took 60 to 90 days to provide information, which was sometimes inaccurate and incomplete (eg, no images and test results). Historical medical records were also difficult to obtain if the physician or the health professional had retired. Another challenge that emerged during phase I was difficulty recruiting participants, potentially due to a lack of technological abilities, time commitment required to learn the system, fear of data security breaches, and lack of internet connectivity (especially in rural areas).

#### Phase II

**Figure 1:**
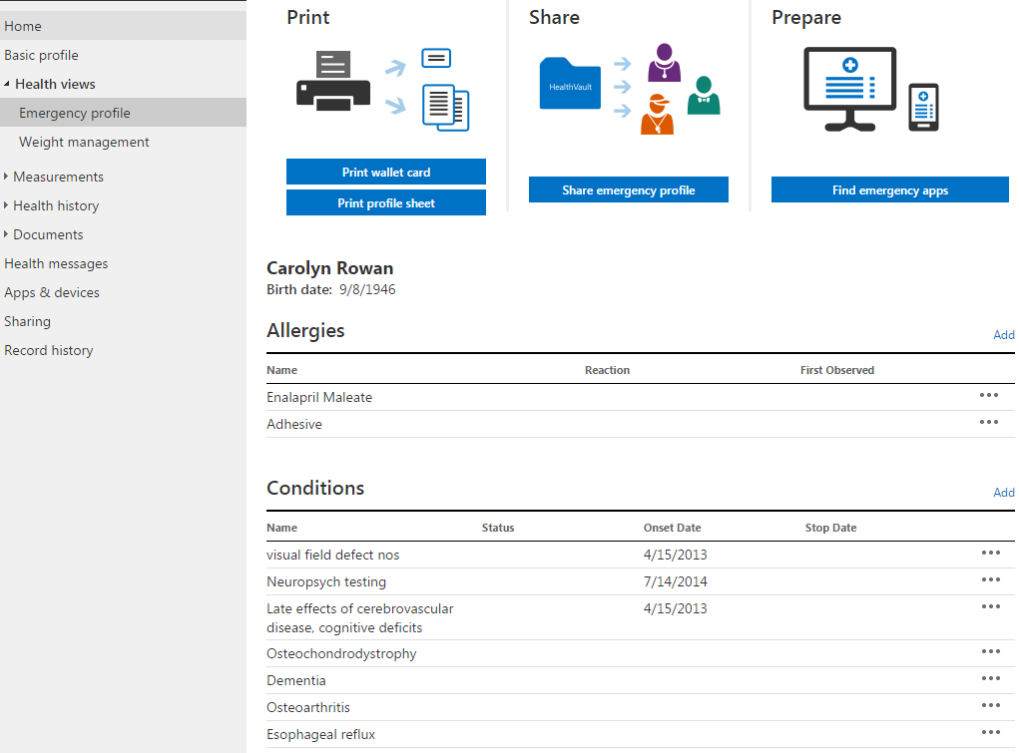
Example Personal Health Record for Persons with Alzheimer Disease or Related Dementia and Their Family Caregivers informational screen—emergency profile.

**Figure 2:**
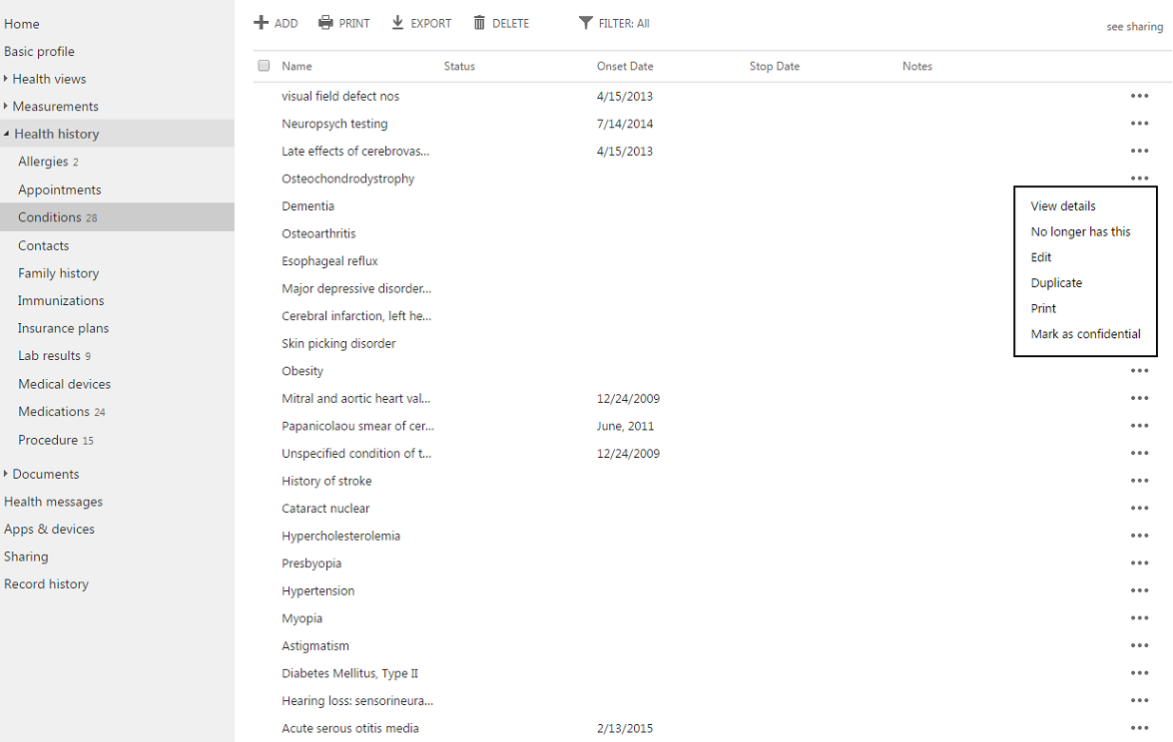
Example Personal Health Record for Persons with Alzheimer Disease or Related Dementia and Their Family Caregivers informational screen—health history.

At the conclusion of phase I [34], the research team partnered with a local developer to test their PHR platform (Alska) for family caregivers of persons with ADRD using the same protocol but with increased attention to recruitment, technological assistance, and obtaining medical information. The phase II platform includes many of the basic features as the platform in phase I (eg, stores demographics; extensive health history, including conditions, immunizations, and test results; and health provider message notifications), with the additional ability to authorize other caregiver users for shared access (Figures 1 and 2) [35].

##### Attention to Recruitment

We adapted our recruitment strategy to address the limitations and challenges of phase I. In phase I, a study counselor identified and recruited family caregivers of persons with ADRD from a local dementia caregiver registry at the University of Minnesota. Phase II enhanced recruitment through more targeted communities and social network outreach. The phase II evaluation also leveraged the social networks of the PHR developer’s president/founder, a former health care advocate and employee of Minnesota’s Office Inspector General, to recruit participants.

##### Technological Assistance

Following the enrollment procedures, the PHR developer created a web-based profile for the ADRD caregiver on the PHR system and scheduled an in-person meeting for a hands-on tutorial at the caregiver’s home or on campus. In the meeting, the PHR developer worked with participants to link their profiles to health care providers, other family members, and local community services. The PHR developer was active in troubleshooting participants’ use of the system and encouraged usage with biweekly email and telephone prompts.

##### Obtaining Medical Information

The expertise of phase II PHR developers and the president/founder helped to actively obtain the care recipient’s medical information. They served as a key liaison between caregivers and providers throughout the study, particularly to obtain health records of persons with dementia when the caregiver was legally authorized to do so. Working directly with the PHR developer also enabled responsive changes in the software to create more flexible data collection systems. It must be noted that for the phase II platform, a PHR does not need to be attached to an EHR to be used by the provider. If providers gave the necessary permission and access to care recipients’ health care data (either via electronic or paper records), this could be entered manually or automatically, depending on record format, into the PHR for use by the caregiver, other individuals, or health professionals, the caregiver could invite the PHR.

### Procedure

After the initial screening for participant eligibility, informed consent from the caregiver and verbal assent from the person living with ADRD (where appropriate) were obtained, and the baseline survey was completed by the caregiver. Next, the participant met with the PHR developer to initialize use with the PHR and to familiarize them with the platform (refer to the *Technological Assistance* section).

### Inclusion Criteria

**Figure 3:**
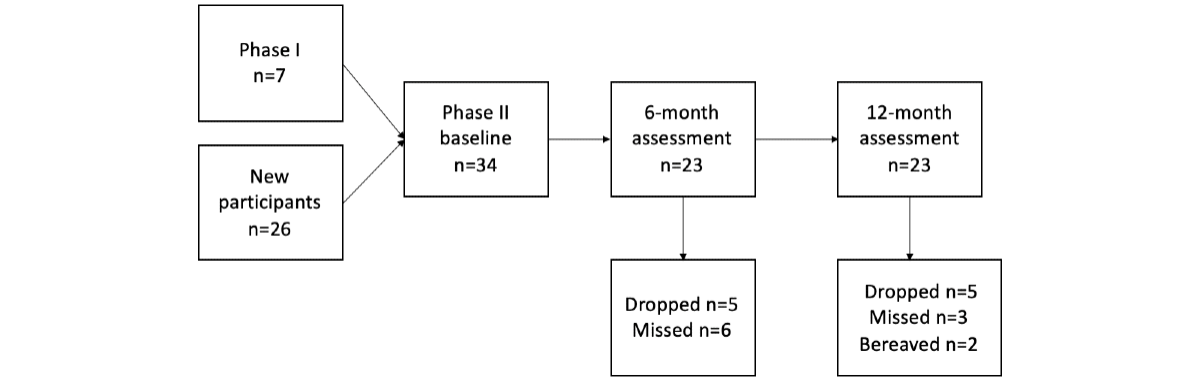
Participant enrollment and engagement flow.

Inclusion criteria were as follows: (1) the care recipient had a physician diagnosis of ADRD; (2) the family member self-identified as someone who provided help to the person with ADRD because of their cognitive impairments; (3) the family caregiver indicated a willingness to use PHR-ADRD for care coordination purposes and access to an internet connection; and (4) the family caregiver provided at least 12 hours of in-person care per week to the person with ADRD at home, in an independent living setting, or in assisted living. As the PHR-ADRD system, surveys, and interviews were all in English, the sample was restricted to English-speaking participants. Some of the interested phase I participants were enrolled in the final evaluation. The participant study flow and involvement are depicted in Figure 3.

Phase II caregivers were surveyed at baseline, 6 months, and 12 months regarding their well-being outcomes and physician interactions. Monthly surveys focused specifically on their use of the PHR-ADRD system in the past month. Both quantitative and qualitative data were collected regarding the use of PHR-ADRD (N=34). At the end of the phase II study, a subsample of participants (n=8) was interviewed using a semistructured interview protocol. In addition, data were collected on provider interaction quality and ADRD caregiver appraisals of their care situation, including self-efficacy and burden.

### Measures

The evaluation of PHR-ADRD is grounded in well-established conceptual models of decision making and dementia caregiving. Encouraging patients of all ages to take an active role in their medical decisions is a keystone of modern practice [36-40], which is encapsulated in the shared decision-making model. This model is proposed to help patients make better clinical decisions and is premised on the belief that good decisions require time, structure, and adequate information [41,42]. In addition, outcome measures were informed by the stress process model, which suggests a mechanism of *proliferation* where the emotional stress of care provision to a person with dementia (the primary stress) spreads to other life domains, which then may negatively influence the caregiver’s mental or physical health and the care recipient’s institutionalization [43-46]. Psychosocial resources or formal service use may help stem stress proliferation and protect dementia caregivers from negative outcomes.

### Context of Care

Baseline variables included demographics of the caregivers and persons with ADRD. Variables specific to the person living with ADRD include time since they saw a doctor for memory problems, living arrangements, and their Medicaid status (Table 1).

**Table 1:** Baseline demographics of caregivers and care recipient dyads (N=34).

Variables			Total		Unfavorable (n=7)		Neutral (n=9)		Favorable (n=8)		Not engaged (n=10)		*P* value^a^
**Caregiver**													
	CG^b^ age (year), mean (SD)	65.4 (12.6)		68.7 (12.9)		62.9 (10.3)		63 (11.2)		67.4 (16.3)		.76	
	CG female, n (%)	24 (71)		3 (43)		7 (78)		7 (88)		7 (78)		.24	
	CG white, n (%)	33 (97)		7 (100)		9 (100)		8 (100)		9 (100)		—^c^	
	CG married, n (%)	28 (82)		7 (100)		8 (89)		6 (75)		7 (78)		.51	
	CG living children, mean (SD)	2.6 (2.2)		2.9 (2.0)		2.2 (1.4)		2.8 (2.7)		2.6 (2.8)		.95	
	CG bachelor’s degree or higher, n (%)	31 (91)		7 (100)		8 (90)		6 (75)		10 (100)		.23	
	CG above median income, n (%)^d^	16 (47)		3 (43)		5 (56)		4 (50)		4 (44)		.95	
	CG employed, n (%)	14 (41)		3 (43)		5 (56)		3 (38)		4 (40)		.88	
**Care recipient**													
	CR^e^ age (years), mean (SD)	77.6 (9.7)		76 (1.5)		73.4 (10.0)		84.1 (7.4)		77 (9.3)		.14	
	CR female, n (%)	17 (50)		5 (83)		2 (22)		6 (75)		4 (44)		.06	
	CR white, n (%)	32 (94)		6 (100)		9 (100)		7 (100)		9 (100)		—	
	CR married, n (%)	21 (62)		4 (67)		7 (78)		3 (38)		7 (7)		.34	
	CR living children, mean (SD)	3.2 (2.1)		3 (1.3)		2.2 (1.6)		4.4 (2.7)		3.4 (2.2)		.22	
	CR bachelor’s degree or higher, n (%)	23 (68)		2 (33)		6 (67)		4 (50)		8 (80)		.27	
	CR above median income, n (%)	23 (68)		3 (50)		6 (67)		7 (88)		7 (78)		.45	
	CR activities of daily living, mean (SD)	1.5 (0.5)		1.6 (0.5)		1.4 (0.6)		1.5 (0.5)		1.5 (0.5)		.97	
	CR instrumental activities of daily living, mean (SD)	1.9 (1.0)		1.6 (1.1)		1.7 (1.0)		2 (1.2)		2.2 (0.6)		.50	
	CR RMBPC^f^ frequency, mean (SD)	24.1 (0.1)		2.3 (0.4)		2.3 (0.6)		2.7 (1.0)		2 (0.2)		.42	
	CR cognitive impairment, mean (SD)	2.9 (0.8)		2.8 (0.9)		2.7 (0.8)		2.6 (0.8)		3.3 (0.6)		.27	
	CR Medicaid, n (%)	5 (15)		1 (17)		1 (11)		2 (25)		1 (11)		.85	
**Dyad**													
	CG is spouse of CR, n (%)	18 (53)		4 (57)		5 (63)		3 (38)		6 (60)		.74	
	CG and CR live together, n (%)	17 (50)		2 (33)		5 (56)		4 (50)		6 (67)		.65	
	CG first noticed CR memory problem, mean (SD), months	64.8 (26.4)		76 (36.1)		56 (32.1)		65.6 (2.6)		65.3 (17.1)		.57	
	CG first helped CR, mean (SD), months	36.2 (24.4)		45 (2.7)		32.3 (26.2)		4.3 (27.2)		31 (27.4)		.65	
	Time (months) since CR seen a doctor for memory problem, mean (SD)	48.3 (25.9)		45.8 (18)		44.1 (31.3)		48.8 (28.9)		52.8 (26)		.91	

^a^*P* values test if characteristic differs by favorability status, Fisher exact chi-square test, or analysis of variance, as appropriate.

^b^CG: caregiver.

^c^—: denotes no statistics were computed because these variables are constant.

^d^≥80,000 for the caregiver and ≥30,000 for care recipient.

^e^CR: care recipient.

^f^RMBPC: Revised Memory and Behavior Problem Checklist.

### Care Recipient Health and Cognitive Status

Care recipient health indicators include their dependence on assistance with *6 activities of daily living tasks* (Cronbach α=.89) [47] and dependence on assistance with *6 instrumental activities of daily living tasks* (Cronbach α=.96) [48,49]. An 8-item scale assessed the intensity of care recipients’ memory losses, communication deficits, and recognition failures at each time point (*cognitive impairment*; Cronbach α=.86) [43,46]. The frequency of *behavioral problems* in persons living with ADRD was measured using the Revised Memory and Behavior Problems Checklist, which lists 30 common problems experienced by persons with ADRD (Cronbach α=.76) [50].

### Caregiver Self-Efficacy, Caregiver Distress, and Resources

An 8-item measure of *caregiver self-efficacy* was used. The 22-item *Zarit Burden Interview* measured caregiver emotional stress (Cronbach α=.92) [51,52]. Two additional measures of subjective stress were used: a 3-item scale assessing caregiver experiences of the involuntary aspects of the caregiving role (*role captivity*; Cronbach α=.78) and a 3-item scale measuring caregivers’ feelings of emotional and physical fatigue (*role overload*; Cronbach α=.83) [43,46]. The 20-item *Center for Epidemiological Studies-Depression* scale measured caregivers’ depressive symptoms (Cronbach α=.85 to .90) [53,54]. An 8-item scale assessed the *socioemotional support* provided to the caregiver by relatives or friends at each time point (Cronbach α=.87) [43,46] See Table 2.

**Table 2:** Baseline caregiver support, self-efficacy, and distress measures, (N=33),

Variables	Total	Unfavorable (n=6)^a^, mean (SD)	Neutral (n=9), mean (SD)	Favorable (n=8), mean (SD)	Not engaged (n=10), mean (SD)	*P* value
Socioemotional support	4.0 (0.8)	4.6 (0.5)	4.0 (0.7)	3.8 (0.7)	3.9 (0.9)	.25
Self-efficacy	3.6 (0.9)	3.7 (1.4)	3.8 (0.6)	3.6 (0.9)	3.4 (0.7)	.73
Burden	2.3 (0.8)	2 (0.8)	2.2 (0.8)	2.2 (0.8)	2.7 (0.8)	.38
Role captivity	2.8 (0.9)	2.4 (1.1)	3.2 (1.0)	2.8 (0.8)	2.7 (0.9)	.48
Role overload	2.8 (1.0)	3.3 (1.0)	3.1 (1.0)	2.6 (1.1)	2.6 (1.0)	.46
Center for Epidemiological Studies-Depression sum^b^	9.4 (7.6)	8.2 (4.2)	9.6 (8.0)	9.4 (1.1)	10 (7.7)	.98

^a^One *unfavorable* participant declined to answer these items.

^b^Center for Epidemiological Studies-Depression scores range from 0 to 30, and higher scores indicate more depressive symptoms.

### Personal Health Record for Persons With Dementia and Their Family Caregivers Feasibility and Utility

Participants were sent a *monthly log* to assess how many days they used the system in the last month, the typical length of time they used it per session, whether they or a provider updated the information on it, and the reason for use. An open-ended question asked why they accessed the PHR-ADRD system in that month. At 6 and 12 months, participants answered via a web-based open- and close-ended survey, 5-point Likert scale *system review* questions about using the PHR-ADRD system to coordinate care for their care recipients. The questions included (1) satisfaction with training, (2) content delivery and support, (3) other factors impacting the use of the PHR-ADRD system, and (4) how PHR-ADRD impacted care coordination across providers. All participants were approached at the conclusion of survey administration to complete a phone-based *semistructured interview* to expand on the utility of the PHR-ADRD system and to identify barriers to use. A full list of the open- and close-ended questions is given in Table 3 and Textbox 1.

**Table 3:** Most recent Personal Health Record for Persons with Alzheimer Disease or Related Dementia and Their Family Caregivers system review checklist by favorability status (N=24).

Variables	Total), mean (SD)	Unfavorable (n=7), mean (SD)	Neutral (n=9), mean (SD)	Favorable (n=8), mean (SD)	*P* value
The PHR-ADRD^a^ was easy to use.^b^	3.4 (1.1)	2.2 (0.4)	3.6 (0.5)	4.0 (1.4)	.01
The information on the introductory screen of the PHR-ADRD was clear to me.	3.6 (1.0)	2.4 (0.5)	3.6 (0.5)	4.5 (0.8)	<.001
The information and screens that I completed on the PHR-ADRD was clear.	3.7 (1.1)	2.2 (0.4)	3.8 (0.5)	4.6 (0.5)	<.001
I was able to understand the options on the PHR-ADRD.	3.6 (1.0)	2.2 (0.4)	3.6 (0.5)	4.6 (0.5)	<.001
The [study counselor], was helpful to me when using the PHR-ADRD.	4.1 (1.1)	3.0 (1.2)	4.3 (0.7)	4.7 (0.8)	.01
I valued having the study counselor present to discuss the service options of the PHR-ADRD.	4.1 (1.2)	3.6 (1.7)	3.6 (0.9)	5.0 (0.0)	.05
After using PHR-ADRD, I was able to find something that looks as though it will meet my needs.	3.1 (1.1)	2.2 (1.0)	2.9 (0.4)	4.1 (0.9)	.001
After using the PHR-ADRD, I was able to find something that looks as though it will meet my relative’s needs.	3.2 (1.0)	2.5 (1.0)	2.9 (0.4)	4.1 (0.9)	.003
My use of the PHR-ADRD led to more positive interactions/communication with my relative’s primary care provider.	3.0 (1.2)	2.6 (0.5)	2.3 (0.7)	4.0 (1.3)	.006
There are time constraints to me being able to use PHR-ADRD (R)^c^.	3.4 (1.4)	4.0 (1.7)	3.5 (1.2)	2.8 (1.3)	.45
I am planning on using the PHR-ADRD regularly.	2.7 (1.3)	1.7 (0.5)	2.3 (0.7)	4.1 (1.1)	<.001
The information provided on the PHR-ADRD was clear and concise.	3.7 (1.0)	2.7 (1.0)	3.8 (0.5)	4.4 (0.8)	.002
I felt lost using the PHR-ADRD (R).	2.3 (1.2)	3.0 (1.4)	2.0 (0.0)	2.0 (1.5)	.32
I wish I would have known about PHR-ADRD sooner.	2.8 (1.2)	1.9 (0.9)	2.8 (0.7)	3.9 (1.2)	.003
After using the PHR-ADRD, I have more confidence providing care to my relative.	2.8 (1.2)	2.2 (0.8)	2.4 (0.7)	3.7 (1.5)	.04
The PHR-ADRD provided me with a sufficient number of options to support me.	3.3 (0.9)	2.6 (0.5)	2.9 (0.4)	4.3 (0.8)	<.001
The PHR-ADRD provided me with a sufficient number of options to support my relative.	3.3 (1.0)	2.8 (0.8)	2.8 (0.5)	4.3 (0.8)	.001
The overall layout, text, and design of the PHR-ADRD is very confusing to me (R).	2.4 (1.3)	4.0 (1.2)	2.0 (0.0)	1.4 (0.5)	<.001
I would be willing to use the PHR-ADRD on my own without [study counselor’s] guidance.	3.2 (1.3)	2.2 (1.5)	3.1 (1.0)	4.3 (0.8)	.009
I would recommend PHR-ADRD to others in a similar situation as I am.	3.5 (1.1)	2.3 (1.1)	3.7 (0.5)	4.5 (0.5)	<.001

^a^PHR-ADRD: Personal Health Record for Persons with Alzheimer Disease or Related Dementia and Their Family Caregivers.

^b^Higher scores indicate more agreement with the item.

^c^R: indicates that lower scores are better. Reverse scores were used for the favorability status allocation.

**Semistructured questions of Personal Health Record for Persons with Alzheimer Disease or Related Dementia and Their Family Caregivers (PHR-ADRD) feasibility and utility.**

**Benefits and ease of use**

- “Was the Personal Health Record for Persons with Alzheimer Disease or Related Dementia and Their Family Caregivers (PHR-ADRD) easy to use?”
- “Why was the PHR-ADRD difficult to use?”

**Functionality**

- “Do you feel the services on the PHR-ADRD worked well for you? Why or why not?”
- “Did the PHR-ADRD help you in interacting with your relative’s primary care provider? Why or why not?”

**Caregiving impact**

- “Did the PHR-ADRD help you feel more confident in providing care for your relative? Why or why not?”
- “Do you think the PHR-ADRD has any effect on how you care for your relative?”

**Other**

- “Please add any other ways that the PHR-ADRD has been helpful to you or how you feel the PHR-ADRD could be improved.”

### Analysis

A total of 24 participants completed at least one PHR-ADRD *system review checklist*. The mean of the participants’ latest PHR-ADRD system review Likert sum score (1=strongly disagree, 2=disagree, 3=neutral, 4=agree, and 5=strongly agree) was recoded into an overall favorability score by the top, middle, and bottom third percentiles, using 33.3% and 66.7% cutoff points. These corresponded to <3.03=unfavorable, 3.03-3.58=neutral, and >3.58=favorable groupings with group means 2.19 (SD=0.65), 3.22 (SD=0.14), and 4.31 (SD=0.61), respectively. The recoding procedure resulted in 7 unfavorable participants, 9 neutral participants, and 8 favorable participants with statistically significantly different checklist mean scores (*F*_2,21_=33.09; *P*<.001). Ten more participants were coded as *not engaged* because they either were missing all follow-up surveys (n=5), left answers blank (n=2), or filled in all *not applicable* (n=3) for the PHR-ADRD system review checklist.

Baseline descriptive means and counts were compared among the unfavorable, neutral, favorable, and not engaged participants (analysis of variance [ANOVA] or chi-square analyses as applicable) to identify the characteristics and use experiences of those who liked or disliked the PHR-ADRD system. The PHR-ADRD system review checklist item mean scores were compared among the participants with checklist data (ANOVA). Analyses were performed using SPSS 25 (IBM Corp).

The brevity of comments on the PHR-ADRD monthly use questionnaire, the open-ended system review questions at the 6- and 12-month follow-up, and the semistructured interviews precluded a traditional in-depth qualitative thematic analysis. Instead, two coders read all qualitative data and selected quotes that provided insights into the quantitative patterns and suggested opportunities for future research.

## Results

### Sample Characteristics

A total of 34 caregiver-care recipient dyads were included in the survey. The baseline mean caregiver age was 65.4 (SD=12.6) years, about 70% (24/34) were female, nearly all had a bachelor’s degree or higher (31/34, 91%), and all were white. The baseline mean care recipient age was 77.6 (SD=9.7) years, 50% (17/34) were female, a majority had a bachelor’s degree (23/34, 68%), and all were white. Only 15% of the care recipients were on Medicaid. Half of the dyads were spouses (18/34, 53%;) or living with each other (17/34, 50%). Caregivers had been helping their care recipient for an average of 36.2 months (or approximately 3 years; mean 35.2, SD 24.4 months). Other demographic characteristics are shown in Table 1. In this study, caregivers were at the high end of socioemotional support and self-efficacy and reported low levels of burden, role captivity or overload, and depressive symptoms (Table 2).

### Characteristics by Personal Health Record for Persons With Dementia and Their Family Caregivers Favorability

None of the baseline characteristics were significantly related to participants’ degree of favorability (unfavorable, neutral, favorable, or not engaged) toward PHR-ADRD, as shown in Table 1. Furthermore, no statistically significant differences between the favorability status groups were indicated at baseline, 6-month, or 12-month follow-up measures of social support, self-efficacy, feelings of burden, role captivity, role overload, or depressive symptoms. Correlations and chi-square analyses using participants’ continuous mean PHR-ADRD review checklist utility scores yielded similar nonsignificant results. Altogether, this suggests that none of the quantitatively measured variables were related to PHR-ADRD experiences.

Participants who failed to engage with the technology suggested that they would have been more likely to view the technology favorably had their living arrangements or caregiving context required more coordination across either caregiver or geographic location. For example, one caregiver explained that the technology would be most useful in cocaregiving situations where multiple caregivers share responsibility for providing care. In this context, the caregiver thought PHR-ADRD would be especially useful when caregivers are not living in the same city. Another female participant felt that the tool would be beneficial to others but was not useful in her situation because her mother received her health care through the residential care facility where she lived:

I think it would have been really helpful, but we never actually had the opportunity to enter any information because it wasn’t needed, but when I talk to other friends who are having all these family fights and issues because they don’t know what’s going on, or they don’t have access to looking something up on the internet, I just think it would have been so incredibly helpful.

Nonetheless, this observation failed to come to bear in the quantitative data. Statistical analyses showed no difference in favorability or engagement by either living arrangement (*F*_3,33_=1.28; *P*=.73) or spousal status (*F*_3,33_=1.66; *P*=.65).

### Personal Health Record for Persons With Dementia and Their Family Caregivers Feasibility and Utility

There was a statistically significant decrease (t_14_=4.21; *P*=.001) in the overall mean PHR-ADRD review checklist utility scores for the subsample that completed system reviews at both time points. The 12-month review checklist had a mean score of 3.84 (SD=0.74) and the 3-month checklist had a mean of 3.22 (SD=1.06) for a mean difference of –0.46 (95% CI −0.70 to −0.23).

Qualitative interviews provide insight into why PHR-ADRD may have been useful for some caregivers and less useful for others. One interview respondent appreciated that the technology organizes everything in one place. The caregiver explained:

It’s one place shopping. Everything is there for me. When we’ve gone to the hospital, all I’ve had to do is print out, or take my computer with me, his medications, his previous hospitalizations, all of his doctors contact numbers, the site is very easy to use...There’s also a place to store all of the legal documents, his power of attorney, his medical directives, his POLST forms, and that’s very helpful. You don’t have to grab 100 papers if you need to use any of those documents.

She went on to explain that PHR-ADRD also helped her husband stay engaged in his care:

I think he also likes the fact that when we need to go to the hospital, or some kind of medical thing, that he can tangibly hold on to the papers and feel like he’s also part of the discussion.

Other caregivers found the tool useful for organizing medications, to-do lists, and appointments.

Despite these advantages, other users felt as though the technology was redundant and needlessly complex. One caregiver said that it was easier for her to call her adult children to provide updates rather than enter updates into PHR-ADRD. She explained that the system’s alert feature alerted users of updates but did not specify what was updated. This left users to search through PHR-ADRD, looking for what had been updated. She elaborated:

I would put something in and they would get an alert, but they didn’t know where I had put something in, under which category, and they didn’t take the time to search out where I had put it.

Several caregivers said that they already had access to similar tools (eg, MyChart) through their health care provider and using the PHR-ADRD was redundant.

Individual items were examined using correlations with participants’ overall mean checklist scores and across favorability status groups using the tertile cutoffs (ie, unfavorable, neutral, or favorable) with an ANOVA approach (the *not engaged* participants had no PHR-ADRD checklist scores and so were not included in these comparisons). All correlations were statistically significant with the exception of two items, which were mirrored in the ANOVA analyses. As shown in Table 3, only *I felt lost using the PHR-ADRD* (*R*^2^=0.347; *P*=.12) and *There are time constraints to me being able to use PHR-ADRD* (*R*^2^=−0.218; *P*=.40) were not associated with group status. Participants generally did not feel lost using PHR-ADRD but did feel that time was a barrier to using the system.

Qualitative data echoed our finding that the time to learn and use PHR-ADRD was a barrier. Caregivers noted in open-ended questions that they were too busy to use the PHR-ADRD technology. In addition to the lack of time, this could indicate that the platform was too complicated and not user-friendly. For example, one participant reported that the system had *bugs* and discontinued using the system. In addition to the technology itself, users’ level of comfort with technology is another potential explanation for the low engagement and favorability among some caregivers. According to one caregiver:

I think it’s very worthwhile if you have relatives spread out around either the state, or the United States. I guess one thing that I had a problem with- and this is my fault for not pursuing it- is, because I’m not that computer savvy, I didn’t really know how to enter different reports we got from the doctor. I didn’t know how to put that into the system.

There were no statistically significant differences in PHR-ADRD use and engagement with the system between favorability groups as measured by mean days accessed, minutes spent during each access session, or times updated with health information (either by the CG or by a provider), as reported in the monthly logs (Table 4). The *not engaged* participants were significantly less likely to have filled out the monthly log in the first place (*F*_3,30_=4.88; *P*=.007), which resulted in a lack of data for comparison of the monthly log items.

**Table 4:** Total Personal Health Record for Persons with Alzheimer Disease or Related Dementia and Their Family Caregivers use and log-use descriptives (N=34).

Variables	Total, mean (SD)	Range of responses	Unfavorable (n=7), mean (SD)	Neutral (n=9), mean (SD)	Favorable (n=8), mean (SD)	Not engaged (n=10), mean (SD)	*P* value
Number of monthly logs completed	3.4 (2.8)	0-8	3.6 (2.6)	5.0 (2.6)	4.3 (3.1)	1.0 (1.5)	.007
Total number of days the site was used^a^	8.7 (22.2)	0-96	1.3 (1.5)	3.1 (4.9)	24.5 (38.9)	—^b^	.12
Total minutes of each site visit	26.5 (16.4)	7-70	3.0 (9.0)	21.5 (8.9)	3.1 (27.5)	—	.72
Total number of times the caregiver or the care provider updated the site information	1.0 (1.4)	0-5	0.5 (0.5)	0.9 (1.2)	1.8 (1.9)	—	.23

^a^Not engaged participants were only in the monthly log comparisons and therefore have missing data for the other comparisons.

^b^Missing data.

### Caregiver Provider Interaction

Favorable participants were most likely to agree that the use of the PHR-ADRD system *led to more positive interactions/communication with my relative’s primary care provider*, whereas neutral and unfavorable participants were more likely to disagree (*P=*.006; Table 3). Very few caregivers updated or had their provider update the PHR-ADRD site with their medical information (total number of updates ranged from 0 to 5 over the whole study period), and it did not differ by favorability status (*P*=.22; Table 4).

Qualitative data point to a lack of provider buy-in as a barrier to PHR-ADRD engagement. One caregiver elaborated:

I think it’s a brilliant program. I just think it needs to get started from the hospital/doctor standpoint...I wasn’t able to really use the platform because my doctors and nurses and pharmacists didn’t use it.

Another explained the barriers to using the platform and pointed to systemic barriers in the health care system:

I engaged several providers but ultimately hit a dead end each time. They won't share information directly with [the PHR-ADRD system], and they won't access the information even if they are entered by other healthcare providers. The responsibility falls onto the caregivers' shoulders to specifically request the information each time...This I find overly burdensome and that is why I finally gave up.

In all, participants were frustrated with entering their care recipients’ medical information into the PHR-ADRD system and desired more buy-in from their providers to resolve this issue. Even favorable participants only updated or had their provider update their information about twice over the 12-month study period.

## Discussion

### Summary of Results

The PHR-ADRD system was neither extensively used nor favorably regarded by a majority of caregivers in the study, even with the PHR developer support and the use of a more interactive and flexible PHR platform. In particular, about one-quarter of the enrolled participants were not engaged with the PHR-ADRD system to the extent that they did not fill out the system review checklist at any follow-up. Still, the users who did like the system (ie, the favorable group) consistently had positive reactions to all aspects of it, as seen in the items listed in Table 3.

Although a majority of older adults are interested in technological solutions to assist in caregiving [55], the dissemination and actual use of these tools has been less successful due to issues with web technology such as ease of use, availability of support, and computer literacy for both users and clinicians [56]. These issues seemed consistent in this PHR-ADRD study despite the system being designed with the goal of reducing the time needed to manage health information (eg, provider message notification capability and record access) and technology support provided by the developer. Participants still felt that there were time constraints and reported technological issues as barriers to effectively using the PHR-ADRD system. These findings align with a recent study that identified several ways in which another similar internet-based medical management tool was perceived as difficult to use: caregivers were reluctant to add another management tool to their already busy day-to-day activities, found the system itself difficult to use in terms of cognitive workload, and reported the system’s tools to be of limited dynamic functioning [57]. Although this study’s participants did not feel particularly lost using the PHR site, systems designed for ADRD caregivers need to pay extra attention to user interface design to equitably reduce cognitive and time burdens for users from all technological backgrounds [58].

Furthermore, concerns about privacy and confidentiality among ADRD caregivers and their care recipients may have limited the success of the PHR-ADRD tool [59]. A recent AARP survey found that about one-third of respondents did not trust health care companies to keep personal data secure on the web [55]. To allay these privacy issues, this project enlisted the help of the PHR developer to call or meet with participants to discuss their concerns. However, not all participants experienced the same benefits from this contact, as the helpfulness of this was perceived differently by favorability status. Building in administrative or advisory support for PHR systems that meet the needs of all users will increase the likelihood of favorability reception and may alleviate concerns about privacy. In addition, PHR developers need to ensure privacy and confidentiality through high-quality security, employee training, and system audits [28].

Participants indicated that they may not have particular use for this kind of shareable medical platform. Over 93% (17/18) of the spousal caregivers in this study lived with their care recipient at baseline and so likely share less of the caregiving responsibilities with other family members. Family members who share caregiving responsibility with others may benefit more from the ability to manage and exchange medical data [18,60]. The qualitative data suggested that the tool may be more useful for those coproviding care and those whose care recipients are not residing in a long-term care facility. However, post hoc ANOVA analyses showed no difference in favorability or engagement groups by either living arrangement or spousal status.

In the future, systems such as PHR-ADRD may be better received as more services are digitized, internet access is more universal, and the aging population becomes more technologically literate. EHRs are now used by more primary practices, and broadband penetration is making access to high-speed internet a reality for an increasing number of people, making such internet-based platforms for sharing medical information potentially more feasible [61,62]. Nonetheless, this study suggests there are still ongoing practical and translational issues regarding provider buy-in and the transfer of medical data into web-based systems such as PHR-ADRD, particularly third-party platforms external to the health care system. Qualitative data indicate that lack of provider use and difficulty in sharing data across health care systems was a barrier to the usefulness of PHR-ADRD. Negative provider PHR-related attitudes, extra work, and lack of reimbursement are potential reasons for the lack of provider buy-in and EHR facilitation. Provider buy-in may also allay potential privacy and security concerns [63]. This will have to be addressed even as the market moves into the development of user-friendly mobile phone apps [64].

### Strengths

This was a multiphase, mixed methods approach to testing the PHR-ADRD system, an internet-based medical health platform aiming to serve caregivers of persons living with dementia. This study attempted to build on the successes of its pilot phase to improve facets of the research design and PHR-ADRD tool while giving voice to the caregivers (open-ended questions and interviews) with an eye toward continuous development. The PHR-ADRD system itself was developed with a person-centered approach, geared toward shared decision making, and allowed PHR-ADRD caregivers and authorized users access to the medical data stored on the system. By leveraging the network and expertise of the PHR-ADRD developer, the final evaluation was able to recruit a larger sample and make early changes to the software to enhance health data collection within the system. A previous relationship with the PHR-ADRD developer for a limited number of participants did not appear to bias the results of the study, given the diversity of positive and negative reflections on the use of the system.

### Limitations

Despite increased outreach efforts and time devoted to recruitment of ADRD caregivers, this study still fell short of its original recruitment goal, both in terms of sample size (only 34 instead of 50) and diversity (all white participants). The general lack of diversity among older persons in Minnesota, where this study took place, limited recruitment in this regard. The nature of PHRs themselves may have limited recruitment and engagement as they currently require providers or caregivers to manually enter EHRs and do not appear as novel as other technology-based interventions such as in-home sensors or robotic aids. The small sample size may have limited the discovery of statistically significant differences to corroborate the qualitative findings. However, these exploratory analyses did demonstrate barriers that should be overcome before proceeding to a larger trial. The lack of significant findings should not be taken as definitive evidence that relationships do not exist. In addition, the follow-up and engagement of the participants was limited. Participant contact logs should be kept to evaluate whether technological and administrative support can improve PHR-ADRD feasibility and utility. Finally, PHR use information was limited to self-report by the participant, which may have resulted in reporting bias.

### Conclusions

The technological literacy of some participants, inherent complexity of a web-based PHR system, and lack of provider buy-in were considerable barriers to a majority of participants favorably engaging with this study’s PHR-ADRD system. Furthermore, the PHR-ADRD system may not have been useful for those living with and providing sole care to their care recipient. Even so, a third of the participants found many facets of the system to be beneficial, such as medical document consolidation and portability. Future PHR-ADRD development and adoption efforts should focus on reducing user interface complexity, increasing technological support, and improving provider buy-in and health record access so that these rapidly emerging dementia caregiver support tools can exert positive, meaningful benefits for people living with ADRD and their family caregivers.

## Abbreviations

ADRD: Alzheimer disease and related dementias
ANOVA: analysis of variance
EHR: electronic health record
PHR: personal health record
PHR-ADRD: Personal Health Record for Persons With Alzheimer Disease or Related Dementia and Their Family Caregivers
